# Contextual variability in under-diagnosed cardiometabolic disease and cognitive vulnerability among populations at high risk for Alzheimer’s disease and related dementias

**DOI:** 10.1038/s44400-026-00099-3

**Published:** 2026-06-19

**Authors:** Nwanyieze Ngozi Jiakponnah, Joseph Curran, Tamlyn Watermeyer, Jasmit Shah, Litha Musili, Stanley Onyango, Benard Aliwa, Andy Mackelfresh, Omonigho Michael Bubu, Chiadi Onyike, Ozioma Okonkwo, Zul Merali, Rufus Akinyemi, Timothy Hughes, Mansoor Saleh, Melissa Petersen, Karen Blackmon, Adesola Ogunniyi, Hugh Hendrie, Chinedu Udeh-Momoh

**Affiliations:** 1 Department of Epidemiology and Prevention, Wake Forest University School of Medicine, Winston-Salem, NC USA; 2 School of Psychology, University of Northumbria, Newcastle, UK; 3 School of Neurological and Cardiovascular Sciences, University of Edinburgh, Edinburgh, Scotland UK; 4 Imarisha Center for Brain Health and Aging, Brain and Mind Institute, Aga Khan University, Nairobi, Kenya; 5 University of North Carolina, Charlotte, NC USA; 6 Departments of Psychiatry, Neurology & Population Health, NYU Grossman School of Medicine, New York, NY USA; 7 Division of Geriatric Psychiatry and Neuropsychiatry, Johns Hopkins University School of Medicine, Baltimore, USA; 8 School of Medicine and Public Health, University of Wisconsin-Madison, Madison, WI USA; 9 Neuroscience and Ageing Research Unit, Institute for Advanced Medical Research and Training, College of Medicine, University of Ibadan, Ibadan, Nigeria; 10 Department of Neurology, University College Hospital, Ibadan, Oyo Nigeria; 11 Department of Internal Medicine, Section on Gerontology and Geriatrics Medicine, Wake Forest University School of Medicine, Winston-Salem, NC USA; 12 Cancer Research Unit, Aga Khan University, Nairobi, Kenya; 13 Institute for Translational Research, University of North Texas Health Science Center, Fort Worth, TX USA; 14 Geisel School of Medicine, Dartmouth University, Hanover, NH USA; 15 Indiana University School of Medicine, University of Indiana, Indianapolis, IN USA; 16 Division of Clinical Geriatrics, Center for Alzheimer Research, Care Sciences and Society (NVS), Karolinska Institutet, Stockholm, Sweden; 17 Sheffield Institute for Translational Neuroscience, Sheffield University, Sheffield, UK

**Keywords:** Biomarkers, Diseases, Neurology, Neuroscience, Risk factors

## Abstract

Underdiagnosis of cardiometabolic risk factors (CMRFs) may represent an unrecognised biological pathway contributing to dementia risk; yet remains poorly characterised in African and African diaspora populations. We quantified the prevalence and determinants of underdiagnosed hypertension and abnormal glycaemia across four cohorts comprising up to 7000 adults aged ≥40 years from Nigeria, Kenya, and The United States: Indianapolis, and North Texas. Underdiagnosis was defined as absence of self-reported diagnosis despite elevated systolic blood pressure ( ≥130 mmHg) or fasting blood glucose ( ≥100 mg/dL). Cohort-stratified analyses examined demographic, socioeconomic, cognitive, Alzheimer’s genetic, and blood-based biomarker correlates. Underdiagnosis was pervasive in African cohorts. Elevated fasting glucose was associated with cognitive impairment in Kenya and North Texas, while severe hypertension and diabetes were linked to Alzheimer’s disease-related biomarkers [pTau217/181, NFL and Aβ42/40] in North Texas (*all p* ≤ *0.05*). These findings identify context-specific diagnostic gaps in populations at high dementia risk and highlight cardiometabolic detection as a mechanistic target for prevention.

## Introduction

Dementia is a growing global health challenge, with prevalence projected to rise significantly among individuals of African descent^1,2^. This trajectory parallels the increasing burden of cardiometabolic risk factors (CMRFs), including hypertension, diabetes, and hyperlipidemia, which contribute to dementia through vascular and metabolic pathways that impair cerebrovascular integrity and accelerate neurodegeneration^3–5^. The 2024 *Lancet* Commission on dementia identified CMRFs as central components of the 14 modifiable risk factors that together account for approximately 45% of global dementia cases, indicating the substantial contribution of cardiometabolic pathways to dementia risk worldwide^6^. These cardiometabolic contributors are also increasingly recognized as dominant drivers of the expanding dementia burden in many parts of Africa^1^.

By 2030, in Sub-Saharan Africa, CMRFs including hypertension, type 2 diabetes mellitus, obesity, and hyperlipidemia are projected to become leading causes of morbidity and mortality, surpassing infectious diseases as the primary causes of death in the region^7,8^. Similarly, among diasporic African populations, such as African Americans, CMRFs are more prevalent than in other racial groups^4^, emphasizing the relevance of cardiometabolic health to dementia risk across both indigenous and diaspora African populations. However, the true scale of this risk may be significantly underestimated due to critical gaps in subjective awareness and clinical diagnosis. Indeed, current evidence indicates that an estimated 73% of adults with hypertension and 62.5% of those with diabetes in Sub-Saharan Africa remain undiagnosed^9,10^. This suggests that the substantial yet often overlooked burden of undiagnosed cardiometabolic disease in the region could be a potential hidden driver of the emerging dementia crisis. In the United States, African American adults with hypertension and diabetes are also more likely to be unaware of their condition compared to other racial groups, reflecting persistent disparities in screening and diagnosis^11^. Collectively, these findings point to a widespread pattern of underdiagnosis across both indigenous and diaspora populations. This trend is driven largely by inequities in healthcare access, diagnostic practices, and wider social determinants of health^4,5,12^.

Despite this growing evidence base, little is known about how underdiagnosis of CMRFs varies across demographic and contextual settings. Factors such as age, sex, educational attainment, socioeconomic status, and geography influence awareness of disease symptoms, healthcare-seeking behavior, and adherence to prevention and treatment^9,13^. Cultural, structural and health-system barriers including limited screening resources, fragmented healthcare infrastructure, and competing public health priorities often lead to delayed or missed diagnoses, particularly among older adults^12^. It also remains unclear whether the prevalence of undiagnosed CMRFs differs by cognitive status, such that individuals with cognitive impairment or dementia may experience distinct patterns of underdiagnosis compared with cognitively unimpaired individuals. This under-recognition obscures the true burden of cardiometabolic disease, reduces opportunities for timely prevention, and increases vulnerability to cognitive decline in later life.

Using data from four cohorts enriched with African and African diaspora participants, we analyze differences between self-reported and objectively ascertained measures of dementia-related cardiometabolic factors: hypertension and diabetes, to present the first cross-continental investigation of diagnostic gaps across sociodemographic, cognitive, and geographic factors towards identifying subgroups prone to underdiagnosis. The findings will help guide targeted public health efforts to improve cardiometabolic risk detection and reduce brain health disparities.

## Results

### Cohort Characteristics

Participant demographic, cardiometabolic, and cognitive characteristics by cohort are presented in Table 1 and Supplementary Table S1. Cohorts differed significantly in age, education, cardiometabolic profiles, and cognitive status distributions (all overall *p* < 0.001). Mean age was highest in the Indianapolis (78.2 ± 5.8 years) and Ibadan (76.9 ± 5.8 years) cohorts and lower in the North Texas and Kenya cohorts. Women comprised the majority of participants across all cohorts (58.5%–67.5%). Educational attainment varied markedly, with the lowest levels observed in Ibadan, where over 95% of participants had less than secondary school education, compared with 2% in North Texas.

**Table 1:** Sociodemographic and clinical characteristics of the study cohort stratified by location

Variable	Category	Ibadan	Indianapolis	North Texas	Kenya	*p* value
Age	Total Sample Size	2762	2574	1213	451	
	Mean (SD)	76.93 (5.84)	78.24 (5.76)	63.24 (7.82)	56.63 (13.58)	<0.001
Systolic Blood Pressure	Total Sample Size	2751	2326	1211	444	
	Mean (SD)	151.61 (31.38)	145.65 (22.17)	136.64 (18.58)	134.4 (18.46)	<0.001
Fasting Blood Glucose	Total Sample Size	1235	1471	1132	430	
	Mean (SD)	81.43 (27.64)	113.64 (51.4)	102.26 (39.56)	92.89 (33.27)	<0.001
Sex	Total Sample Size	2762	2574	1213	451	<0.001
	F	1854 (67.13%)	1737 (67.48%)	747 (61.58%)	264 (58.54%)	
	M	908 (32.87%)	837 (32.52%)	466 (38.42%)	187 (41.46%)	
Education	Total Sample Size	2759	2567	1213	446	<0.001
	Below Primary School Education (Less than 6)	2571 (93.19%)	106 (4.13%)	1 (0.08%)	10 (2.24%)	
	Completed Primary School Education (At least 6)	130 (4.71%)	56 (2.18%)	1 (0.08%)	71 (15.92%)	
	Completed Secondary School Education (7 – 12 years)	33 (1.2%)	1719 (66.97%)	323 (26.63%)	365 (81.84%)	
	Post Secondary School Education (13 + )	25 (0.91%)	686 (26.72%)	888 (73.21%)		
Marital Status	Total Sample Size	2762	2571	1209	455	<0.001
	Married	1235 (44.71%)	926 (36.02%)	521 (43.09%)	262 (57.58%)	
	Not Married	1527 (55.29%)	1645 (63.98%)	688 (56.91%)	193 (42.42%)	
Unadjusted Dementia/Cognitive Status	Total Sample Size	562	401	1204	443	<0.001
	CI	231 (41.1%)	154 (38.4%)	342 (28.41%)	26 (5.87%)	
	D	44 (7.83%)	54 (13.47%)	68 (5.65%)	28 (6.32%)	
	N	287 (51.07%)	193 (48.13%)	794 (65.95%)	389 (87.81%)	
Dementia Status	Total Sample Size	562	401	1204	443	<0.001
	D	44 (7.83%)	54 (13.47%)	68 (5.65%)	28 (6.32%)	
	ND	518 (92.17%)	347 (86.53%)	1136 (94.35%)	415 (93.68%)	
Cognitive Status	Total Sample Size	562	401	1204	443	<0.001
	CI	275 (48.93%)	208 (51.87%)	410 (34.05%)	54 (12.19%)	
	NCI	287 (51.07%)	193 (48.13%)	794 (65.95%)	389 (87.81%)	
Hypertension (SBP ≥ 130)	Total Sample Size	2751	2326	1211	444	<0.001
	No	719 (26.14%)	545 (23.43%)	443 (36.58%)	193 (43.47%)	
	Yes	2032 (73.86%)	1781 (76.57%)	768 (63.42%)	251 (56.53%)	
Severe Hypertension (SBP ≥ 180)	Total Sample Size	2751	2326	1211	444	<0.001
	No	2234 (81.21%)	2154 (92.61%)	1189 (98.18%)	436 (98.2%)	
	Yes	517 (18.79%)	172 (7.39%)	22 (1.82%)	8 (1.8%)	
Prediabetic/Diabetic (GLU ≥ 100)	Total Sample Size	1235	1471	1132	430	<0.001
	No	1148 (92.96%)	772 (52.48%)	759 (67.05%)	353 (82.09%)	
	Yes	87 (7.04%)	699 (47.52%)	373 (32.95%)	77 (17.91%)	
Diabetic (GLU ≥ 126)	Total Sample Size	1235	1471	1132	430	<0.001
	No	1203 (97.41%)	1106 (75.19%)	985 (87.01%)	405 (94.19%)	
	Yes	32 (2.59%)	365 (24.81%)	147 (12.99%)	25 (5.81%)	
Self-Report Hypertension	Total Sample Size	2750	2552	1212	162	<0.001
	No	1941 (70.58%)	574 (22.49%)	409 (33.75%)	101 (62.35%)	
	Yes	809 (29.42%)	1978 (77.51%)	803 (66.25%)	61 (37.65%)	
Self-Report Diabetes	Total Sample Size	2762	2566	1211	162	<0.001
	No	2691 (97.43%)	1781 (69.41%)	950 (78.45%)	136 (83.95%)	
	Yes	71 (2.57%)	785 (30.59%)	261 (21.55%)	26 (16.05%)	
APOE4 Status	Total Sample Size	1761	1691	1003	¯	0.004
	E4 Carrier	679 (38.56%)	582 (34.42%)	404 (40.28%)		
	Non-E4 Carrier	1082 (61.44%)	1109 (65.58%)	599 (59.72%)		
Multidimensional Poverty Index (categorized)	Total Sample Size	­­­­­­­­­­­­­­ ¯	¯	­­­­­­­­­ ¯	455	
	Deprived ( ≥ 1)				234 (51.43%)	
	Not deprived				221 (48.57%)	
Socioeconomic Disadvantage Index (SDI)	Total Sample Size	¯	¯	1138	¯	
	Mean (SD)	¯	¯	1.18 (0.92)	¯	

*CI* cognitive impairment, *D* dementia, *NCI* no cognitive impairment, *N* normal cognition, *ND* No dementia.

Notes: Values are mean (SD) or n (%). Global p-values compare cohorts using one-way ANOVA for continuous variables and Pearson’s χ² tests for categorical variables. Sample sizes reflect non-missing observations per variable. Pairwise post-hoc comparisons are provided in Supplementary Table S1.

Cognitive status distributions also differed across cohorts, with dementia prevalence highest in Indianapolis (13.5%) and lowest in Kenya (6.3%). Mean SBP and FBG levels differed significantly across cohorts. Ibadan had the highest mean SBP (151.6 ± 31.4 mmHg), while mean FBG levels were highest in the U.S. cohorts (Indianapolis: 113.6 ± 51.4 mg/dL; North Texas: 102.3 ± 39.6 mg/dL). The Kenyan cohort showed intermediate cardiometabolic values.

### Cardiometabolic risk factors and cognitive status

Cross-sectional associations between hypertension, abnormal glycemia, and cognitive status are shown in Table 2 and Table 3 and Supplementary Tables S2–S3 (for dementia models). SBP ≥ 130 mmHg was not significantly associated with cognitive impairment (*p* > 0.5; Table 2) or dementia (*p* > 0.5; Supplementary Table S2). In a sensitivity analysis, SBP ≥ 180 mmHg was likewise not significantly associated with increased odds of cognitive impairment or dementia diagnosis (*p* > 0.5).

**Table 2:** Associations between hypertension and cognitive status by cohort

Location	Outcome	Predictor	Sample Size	Odds Ratio	95% CI Lower	95% CI Upper	P Value
Ibadan	NCI vs CI	Hypertension (SBP ≥ 130)	556	0.95	0.63	1.41	0.785
Indianapolis	NCI vs CI	Hypertension (SBP ≥ 130)	366	1.00	0.62	1.60	0.993
North Texas	NCI vs CI	Hypertension (SBP ≥ 130)	1202	0.98	0.76	1.27	0.889
Kenya	NCI vs CI	Hypertension (SBP ≥ 130)	436	1.07	0.54	2.15	0.841

*NCI* no cognitive impairment, *CI* cognitive impairment.

Models are cohort-stratified logistic regression models adjusted for age and sex. Odds ratios (ORs) and 95% confidence intervals are shown.

**Table 3:** Associations between abnormal glycemia and cognitive status by cohort

Location	Outcome Contrast	Predictor	Sample Size	Odds Ratio	95% CI Lower	95% CI Upper	P Value
Ibadan	NCI vs CI	FBG ≥ 100	253	1.34	0.51	3.52	0.550
Indianapolis	NCI vs CI	FBG ≥ 100	278	1.18	0.73	1.92	0.501
North Texas	NCI vs CI	FBG ≥ 100	1124	1.44	1.11	1.88	**0.006**
Kenya	NCI vs CI	FBG ≥ 100	423	2.47	1.16	5.27	**0.019**

Models are cohort-stratified logistic regression models adjusted for age and sex.

Abnormal glycemia is defined as fasting blood glucose (FBG) ≥ 100 mg/dL. Odds ratios (ORs) and 95% confidence intervals are shown.

*NCI* no cognitive impairment, *CI* cognitive impairment.

Bolded *p* values are statistically significant at *p* < 0.05.

FBG ≥ 100 mg/dL was associated with increased odds of cognitive impairment in the Kenya cohort (OR = 2.47, 95% CI 1.16–5.27, *p* < 0.05) and the North Texas cohort (OR = 1.44, 95% CI 1.11–1.88, *p* < 0.01; Table 3). In supplementary analysis, FBG ≥ 100 mg/dL was associated with increased odds of dementia only in the North Texas cohort (OR = 2.85, 95% CI 1.05–7.79, *p* < 0.05), which was also observed for FBG ≥ 126 mg/dL (OR = 1.85, 95% CI 1.13–2.32, *p* < 0.01).

### Cardiometabolic risk factors and Alzheimer’s disease-related plasma biomarkers

Associations between CMRFs and AD plasma biomarkers were examined in the North Texas cohort, where biomarker data were available. Supplementary Table S4 shows the distribution of plasma biomarkers and association models are presented in Supplementary Table S5. SBP ≥ 180 mmHg was associated with higher plasma pTau217 (β = 0.63, SE = 0.27, *p* = 0.02), pTau181 (β = 0.60, SE = 0.30, *p* = 0.04), and NfL (β = 0.62, SE = 0.27, *p* = 0.02). Overt diabetes defined by FBG ≥ 126 mg/dL was associated with higher NfL (β = 0.37, SE = 0.09, *p* < 0.001) and showed a trend towards an inverse association with the Aβ42/40 (β = -0.27, SE = 0.14, *p* = 0.06).

### Hypertension underdiagnosis across cohorts

**Fig. 1:**
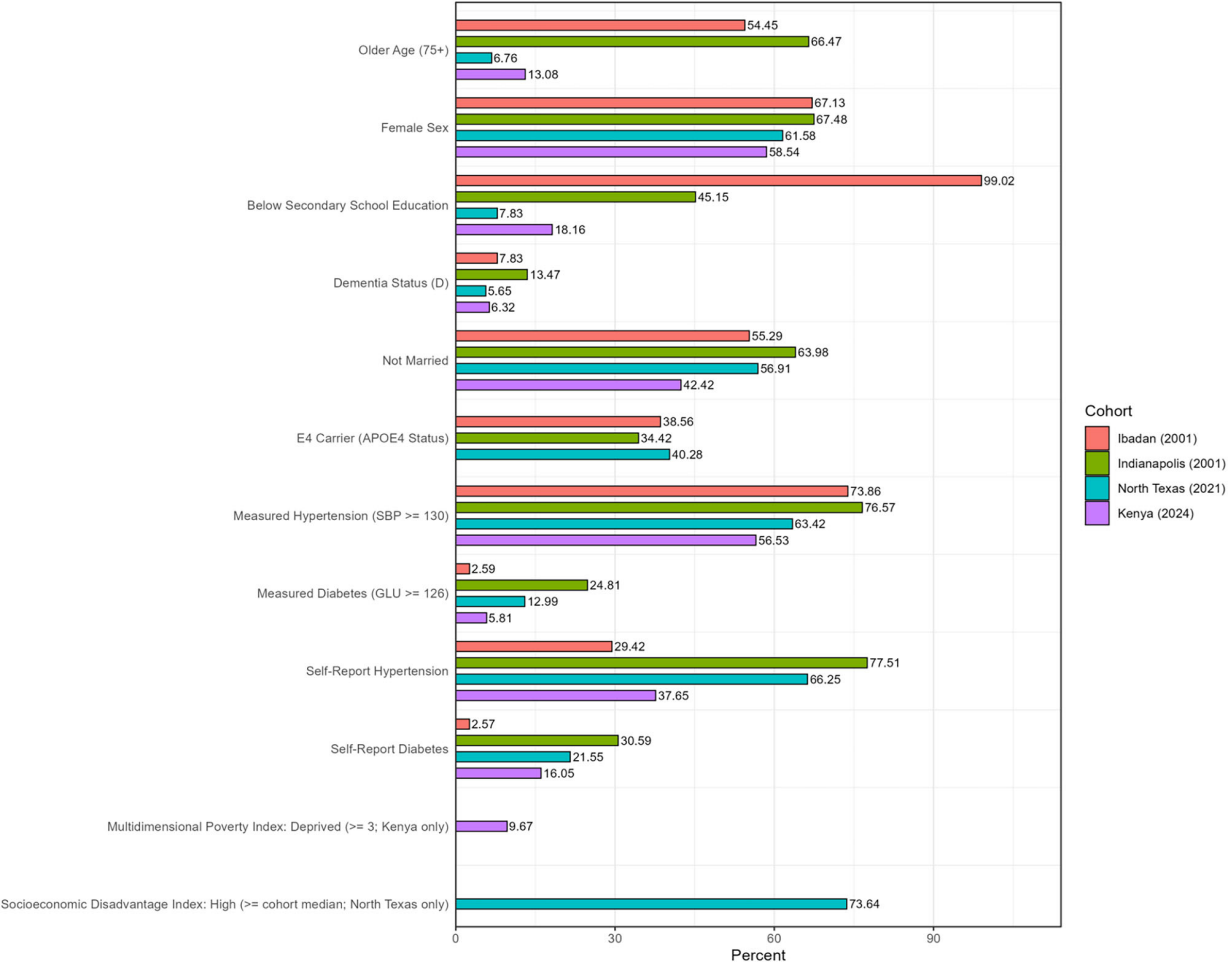
Distribution of demographic, cardiometabolic, and cognitive characteristics by cohort. Values represent percentages within each cohort. Measured hypertension was defined as systolic blood pressure (SBP) ≥ 130 mmHg, severe hypertension as SBP ≥ 180 mmHg, prediabetes as fasting blood glucose (GLU) ≥ 100 mg/dL, and diabetes as GLU ≥ 126 mg/dL. Multidimensional Poverty Index (MPI) categories were defined using a 33% deprivation cut‑off ( ≥ 33% = poor); Socioeconomic Disadvantage Index (SDI) categories were defined using a median split of the five constituent indicators.

The prevalence of hypertension underdiagnosis differed substantially across cohorts (Table 4; Fig. 1). Among participants with objectively measured hypertension, underdiagnosis was highest in Ibadan (67%) and Kenya (53%), and lower in Indianapolis (21%) and North Texas (28%) (overall *p* < 0.001).

**Table 4:** Prevalence of hypertension underdiagnosis by cohort

Measured	Self-Report	Ibadan N	Ibadan %	Indianapolis N	Indianapolis %	Kenya N	Kenya %	North Texas N	North Texas %
SBP ( ≥ 130) = High	Self-Report Hypertension = No	1359	67.18	372	21.06	49	52.69	217	28.29
SBP ( ≥ 130) = High	Self-Report Hypertension = Yes	664	32.82	1394	78.94	44	47.31	550	71.71
SBP ( ≥ 130) = Not High	Self-Report Hypertension = No	573	80.03	148	27.36	51	76.12	192	43.34
SBP ( ≥ 130) = Not High	Self-Report Hypertension = Yes	143	19.97	393	72.64	16	23.88	251	56.66

Underdiagnosis was defined as systolic blood pressure (SBP) ≥ 130 mmHg in the absence of a self-reported hypertension diagnosis.

**Fig. 2:**
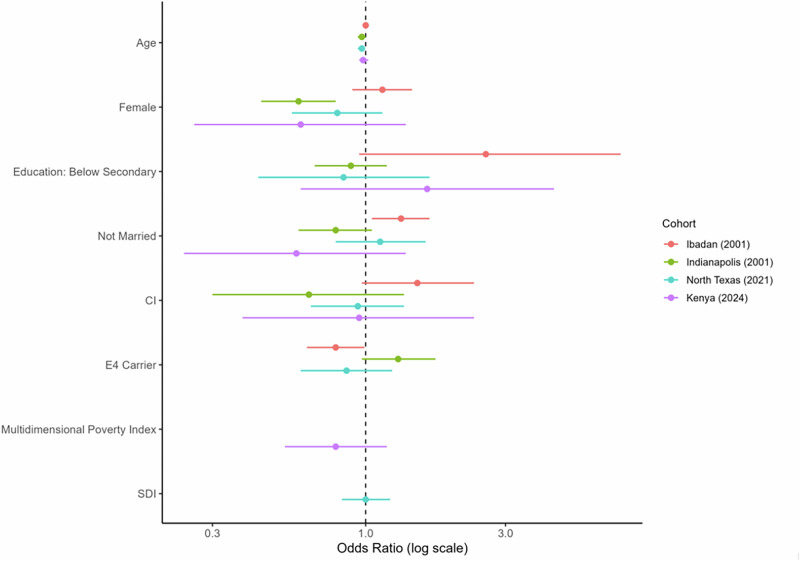
Sociodemographic, cognitive, and genetic predictors of hypertension underdiagnosis by cohort. Note: Points indicate odds ratios (ORs) and horizontal lines 95% confidence intervals from cohort-stratified logistic regression. Hypertension was defined as SBP ≥ 130 mmHg without self-reported diagnosis. Models adjusted for age, sex, education, marital status, cognitive status, and APOE ε4; the Multidimensional Poverty Index was included in Kenya only. The dashed line marks OR = 1.

Cohort-specific predictors of hypertension underdiagnosis are shown in Supplementary Tables S6–S9, with summary effect estimates displayed in Fig. 2. In Ibadan, being unmarried was associated with higher odds of underdiagnosis (OR = 1.32, 95% CI 1.05–1.65, *p* = 0.017). Cognitive impairment showed a trend towards a positive association with underdiagnosis (OR = 1.50, 95% CI 0.97–2.34, *p* = 0.071), while APOE ε4 carriage was significantly associated with lower odds of underdiagnosis (OR = 0.79, 95% CI 0.63–0.99, *p* = 0.043).

In Indianapolis, female sex (OR = 0.59, 95% CI 0.44–0.79, *p* < 0.001) and age (OR = 0.97, 95% CI 0.94–1.00, *p* = 0.025) were associated with lower odds of underdiagnosis, whereas APOE ε4 carriage was associated with higher odds (OR = 1.42, 95% CI 1.03–1.97, *p* = 0.04). No predictors reached statistical significance in the North Texas or Kenya cohorts.

### Abnormal glycemia underdiagnosis across cohorts

Underdiagnosis of abnormal glycaemia (FBG ≥ 100 mg/dL) was common across all cohorts (Table 5), with the highest prevalence observed in Ibadan (93%) and Kenya (61%), and substantial underdiagnosis in Indianapolis (54%) and North Texas (56%) (overall *p* < 0.001). Similar patterns were observed using the stricter diabetes threshold (FBG ≥ 126 mg/dL).

**Table 5:** Prevalence of abnormal glycaemia underdiagnosis by cohort

Measured	Self-Report	Ibadan (n)	Ibadan (%)	Indianapolis (n)	Indianapolis (%)	Kenya (n)	Kenya (%)	North Texas (n)	North Texas (%)
FBG ( ≥ 100) = High	Self-Report Diabetes = No	81	93.10	372	53.53	22	61.11	208	55.76
FBG ( ≥ 100) = High	Self-Report Diabetes = Yes	6	6.90	323	46.47	14	38.89	165	44.24
FBG ( ≥ 100) = Not High	Self-Report Diabetes = No	1116	97.21	662	85.75	104	89.66	680	89.83
FBG ( ≥ 100) = Not High	Self-Report Diabetes = Yes	32	2.79	110	14.25	12	10.34	77	10.17

Underdiagnosis was defined as fasting blood glucose (FBG) ≥ 100 mg/dL in the absence of a self-reported diabetes diagnosis.

**Fig. 3:**
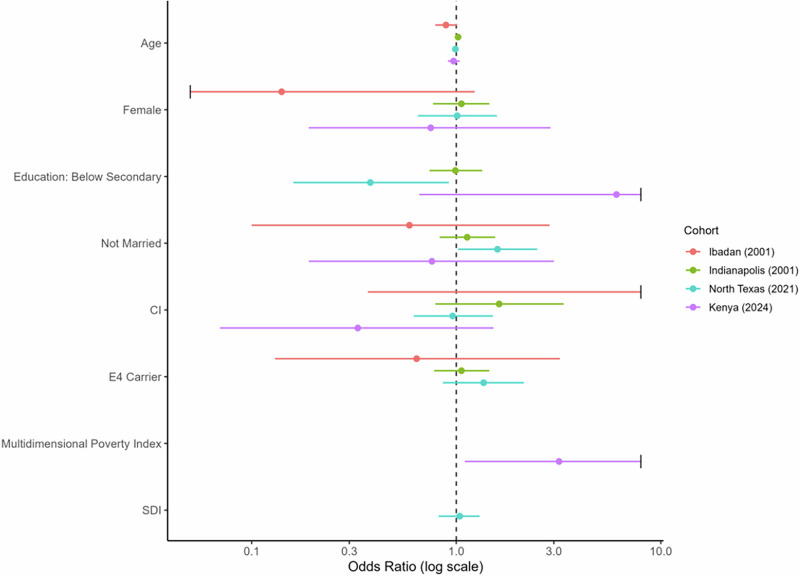
Sociodemographic, cognitive, and genetic predictors of abnormal glycemia underdiagnosis by cohort. Note: Points indicate odds ratios (ORs) and horizontal lines 95% confidence intervals from cohort-stratified logistic regression. Abnormal glycemia was defined as FBG ≥ 100 mg/dL without self-reported diagnosis. Models adjusted for age, sex, education, marital status, cognitive status, and APOE ε4; the Multidimensional poverty index was included in Kenya only. The dashed line marks OR = 1. CI Cognitive impairment, SDI Socioeconomic disadvantage index.

Cohort-specific predictors of abnormal glycaemia underdiagnosis are shown in Supplementary Tables S10–S13, with summary effect estimates presented in Fig. 3. In Ibadan, increasing age showed a trend towards lower odds of underdiagnosis (OR = 0.89, 95% CI 0.78–1.01, *p* = 0.07), while in Indianapolis age showed a trend toward higher odds of underdiagnosis (OR = 1.03, 95% CI 1.00–1.06, *p* = 0.09). In Kenya, higher MPI scores were significantly associated with increased odds of underdiagnosis (OR = 3.18, 95% CI 1.10–9.21, *p* = 0.03).

**Fig. 4:**
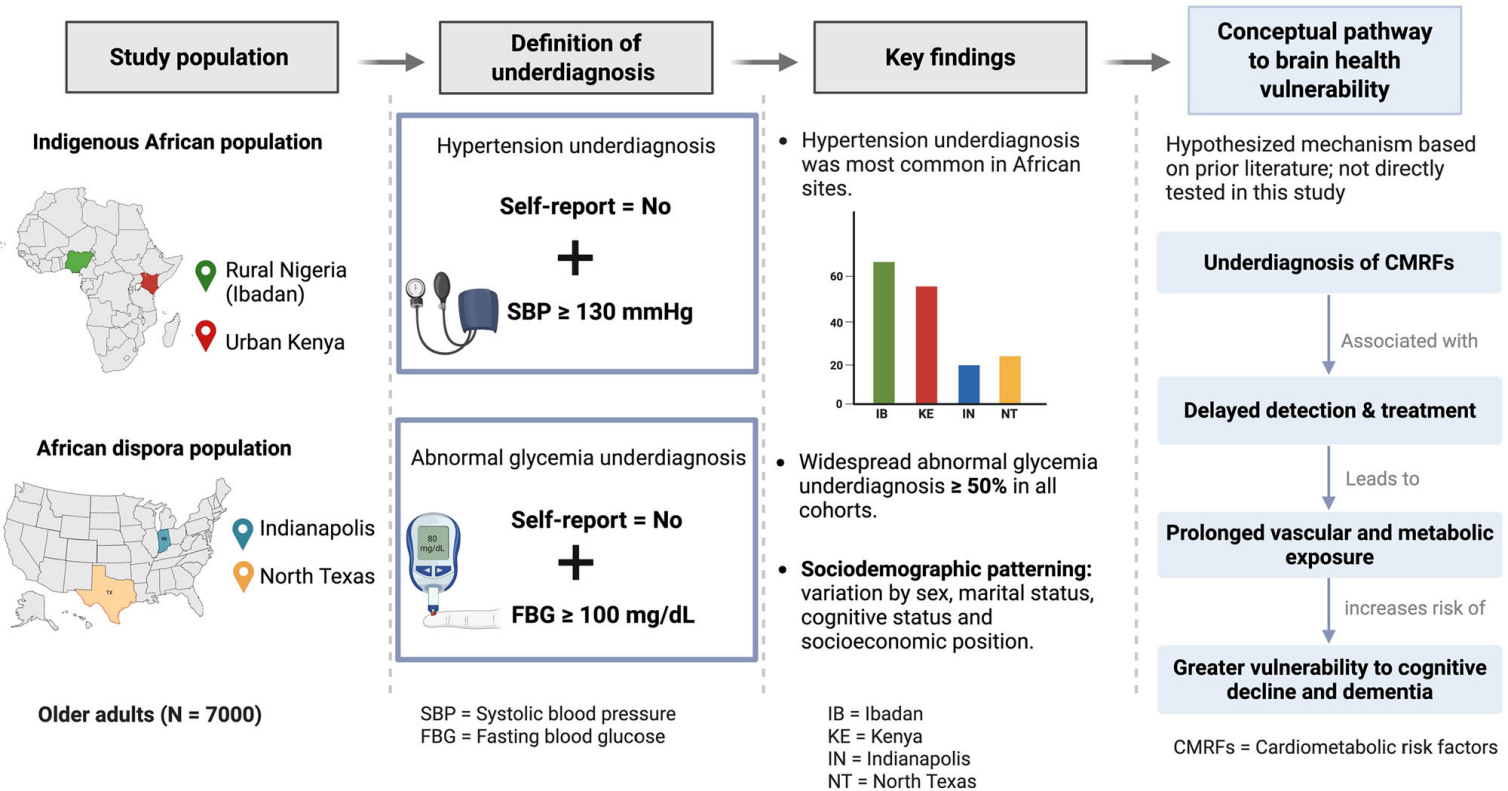
Cardiometabolic risk underdiagnosis and implications for brain health in indigenous African and African diaspora populations. The schematic summarizes study populations, operational definitions of cardiometabolic risk underdiagnosis, and observed prevalence across cohorts. Indigenous African cohorts included Ibadan, Nigeria and urban Kenya; diaspora cohorts included Indianapolis, Indiana and North Texas, United States. Hypertension underdiagnosis was defined as no self-reported prior diagnosis in the presence of systolic blood pressure ≥130 mmHg. Abnormal glycemia underdiagnosis was defined as no self-reported prior diagnosis in the presence of fasting blood glucose ≥100 mg/dL. Bars indicate observed prevalence by cohort. The rightmost panel presents a conceptual framework, based on prior literature, linking cardiometabolic underdiagnosis to delayed detection, prolonged vascular and metabolic exposure, and increased vulnerability to cognitive decline and dementia. These downstream associations were not directly tested in the present study.

In North Texas, having at least a secondary school education (OR = 2.63, 95% CI 1.09–6.36, *p* = 0.03) and not being married (OR = 1.59, 95% CI 1.02–2.49, *p* = 0.04) were associated with higher odds of abnormal glycaemia underdiagnosis. In a sensitivity model including only cognitive status, APOE ε4 status, and a cognitive status × APOE ε4 interaction term, the interaction term was significantly associated with increased odds of underdiagnosis (OR = 3.22, 95% CI 1.24–8.35, *p* = 0.02). Figure 4 summarizes the observed prevalence patterns and sociodemographic correlates of underdiagnosis of CMRFs across cohorts, together with a conceptual link to downstream brain health vulnerability.

## Discussion

In this multi-cohort study of older adults from indigenous African and African diaspora populations, we identified substantial underdiagnosis of hypertension and abnormal glycaemia across all settings, with marked variation by cohort and sociodemographic context. Underdiagnosis was most pronounced in the African cohorts, particularly Ibadan and Kenya, but remained common in U.S. cohorts, indicating that gaps in cardiometabolic disease awareness persist even within health systems with greater clinical infrastructure. Our results suggest that underdiagnosis constitutes a critical and underappreciated barrier beyond what prevalence estimates alone capture for dementia risk reduction across diverse African populations.

Hypertension and diabetes are well-established modifiable risk factors for dementia, particularly when exposure is prolonged or inadequately treated^6,14–17^. Contemporary dementia prevention frameworks, including the 2024 Lancet Commission on dementia, place cardiometabolic health at the center of risk reduction strategies, estimating that a substantial proportion of dementia cases globally are attributable to such modifiable factors^6^. Midlife hypertension and diabetes are robust predictors of late-life cognitive decline and dementia^6,14–17^, and cumulative vascular burden accelerate neurodegeneration and cognitive decline^18^. Evidence further suggests that blood pressure control may reduce the risk of cognitive impairment^18–21^, pointing to the importance of early detection. The present study adds an important dimension to this literature by demonstrating that, in many indigenous African and African diaspora populations, cardiometabolic risk frequently remains undetected. Underdiagnosis thus represents a major missed opportunity for prevention and limits the effectiveness of strategies targeting modifiable risk factors such as blood pressure and glycemic control. In the North Texas cohort, clinically overt cardiometabolic disease (SBP ≥ 180 mmHg and FBG ≥ 126 mg/dL) was associated with AD-related plasma biomarkers (Aβ42/40, pTau, and NfL), indicating that biological markers of brain vulnerability may already be present once disease is established. This suggests that variation in these biomarkers may, in part, reflect downstream effects of uncontrolled cardiometabolic disease rather than primary neurodegenerative processes alone, with important implications for how blood-based biomarkers are interpreted in population studies, particularly in settings where cardiometabolic disease is common and frequently underdiagnosed. This is especially relevant in African settings, where both cardiometabolic disease and dementia are frequently undetected because of diagnostic limitations and structural and health-system constraints, which may complicate the interpretation of biomarker signals in these populations^22,23^.

Patterns of underdiagnosis varied systematically across cohorts, reflecting differences in healthcare access, screening practices, and social context. In Ibadan and Kenya, the majority of participants with objectively measured hypertension or abnormal glycemia were unaware of their condition. These findings are consistent with population-based studies documenting limited screening coverage, low cardiometabolic disease awareness, and constrained diagnostic capacity in many Sub-Saharan African settings^9,24–26^. Structural factors, including multidimensional poverty, limited primary care infrastructure, and competing health priorities, likely contribute to delayed or absent diagnosis in these contexts^23,27^.

Crucially, the observed underdiagnosis was not confined to low-resource settings. In both Indianapolis and North Texas, substantial proportions of participants with abnormal glycemia were unaware of their condition, indicating persistent diagnostic gaps within U.S. health systems. This aligns with prior work showing that African American populations experience disparities in cardiometabolic screening, disease awareness, and continuity of care despite overall healthcare availability^2,4^. These results indicate that underdiagnosis is not solely a function of national income but may reflect complex socio-cultural and health-system barriers shared across the African diaspora population.

Sociodemographic factors further shaped patterns of underdiagnosis of CMRFs in cohort-specific ways. In Ibadan, being unmarried was associated with higher odds of hypertension underdiagnosis. In the U.S. cohorts, older age and female sex were associated with lower odds of hypertension underdiagnosis in Indianapolis, and older age was similarly associated with lower odds in North Texas. For abnormal glycaemia underdiagnosis, a different pattern emerged. In Ibadan, older age and female sex showed non-significant trends toward higher odds of underdiagnosis, whereas in Indianapolis there was a trend toward higher odds with increasing age. In North Texas, lower educational attainment was associated with reduced odds of abnormal glycaemia underdiagnosis, while being unmarried was associated with higher odds. The age-related associations observed for hypertension underdiagnosis in the U.S. cohorts may partly reflect cohort structure and differential engagement with health services across the life course. This is consistent with evidence that older adults tend to have greater health needs and are therefore more likely to utilize healthcare services, leading to increased awareness of their health conditions^28^. The trend toward higher odds of hypertension underdiagnosis among women in rural Ibadan may plausibly reflect gender roles in indigenous African settings that favor patriarchal social structures. This is consistent with prior evidence that older women in rural African settings face distinct barriers to preventive care, including reduced access to screening and lower diagnostic awareness^1^. These observed differences indicate that age-related, gendered, and marital patterns in the underdiagnosis of CMRFs vary across settings and may reflect differences in healthcare access, screening practices, and health-seeking behaviors, reinforcing the need for context-specific approaches to chronic disease detection rather than uniform assumptions about risk or access.

Cognitive status was associated with underdiagnosis in selected cohorts, suggesting that early cognitive changes may complicate disease awareness or engagement with healthcare systems. In Ibadan, cognitive impairment showed a borderline association with hypertension underdiagnosis, while in North Texas, cognitive status interacted with APOE ε4 carriage in relation to abnormal glycemia underdiagnosis. This is consistent with prior studies indicating that cognitive impairment may interfere with symptom recognition, self-report accuracy, and chronic disease management in older adults^29,30^, particularly in settings with limited caregiver or health system support^12,23,27^. This has important implications for dementia prevention. If individuals with early cognitive impairment are less likely to have CMRFs recognized and treated, they may experience prolonged exposure to vascular and metabolic insults during a critical window for risk modification. This could increase dementia vulnerability by delaying interventions that are most effective earlier in the disease course.

We also examined whether APOE ε4 genetic susceptibility modified patterns of cardiometabolic underdiagnosis. Associations varied across cohorts and outcomes, and no consistent direction or significant main effect of APOE ε4 was observed after adjusting for sex, age, education, socioeconomic indicators, and cognitive status. The only exception was in the North Texas cohort, where an APOE ε4 × cognitive status interaction was associated with underdiagnosis of abnormal glycaemia; however, this finding did not replicate in other settings. Across cohorts, the pattern indicates that variability in CMRFs underdiagnosis is more closely tied to social, cognitive, and healthcare factors than to genetic susceptibility, aligning with prior evidence that awareness, diagnosis, and management of cardiometabolic conditions are primarily shaped by social determinants and health system characteristics rather than biological risk alone^31,32^. Although APOE ε4 influences lipid metabolism and cardiometabolic physiology^33^; our findings caution against over‑emphasizing genetic explanations for diagnostic disparities and instead highlight the central role of structural determinants. While APOE ε4 is a well‑established genetic risk factor for dementia, our results indicate that elevated genetic risk does not reliably confer greater likelihood of cardiometabolic screening or detection in clinical or community settings. Consequently, individuals with higher genetic susceptibility may remain exposed to unmanaged vascular and metabolic risks, which has implications for dementia prevention strategies that rely solely on genetic profiling.

From a policy perspective, our findings align with calls to strengthen cardiometabolic detection as a cornerstone of dementia prevention, particularly in African populations. Initiatives such as the AFRICA-FINGERS^34^, and FeMBER-AFRICA^35^ emphasize integrated approaches that combine early detection, community-based screening, and health system strengthening to address modifiable dementia risk factors across the life course.

Our results indicate that without addressing underdiagnosis, even well-established prevention frameworks may fail to reach populations at greatest risk. Improving screening coverage, diagnostic awareness, and follow-up care should therefore be considered essential components of dementia risk reduction strategies in both indigenous African and African diaspora contexts.

This study has several notable strengths. By combining data from three geographically and culturally distinct cohorts, it provides, to our knowledge, the first cross-continental analysis of cardiometabolic underdiagnosis, rather than prevalence, in relation to dementia risk across both indigenous African and African diaspora populations. Integration of objective cardiometabolic measures with self-reported diagnosis enables direct quantification of diagnostic gaps, revealing the extent of hidden morbidity often overlooked in surveillance studies. The sex-disaggregated and context-sensitive analyses further uncover distinct vulnerability profiles, highlighting the need for tailored prevention strategies. Although cross-sectional, the study establishes a foundation for longitudinal investigation within established cohorts to clarify how early detection of cardiometabolic disease may mitigate cognitive decline.

Limitations should be acknowledged. The cross-sectional design precludes causal inference, and differences in measurement protocols across cohorts may contribute to heterogeneity. Cognitive status was determined using cohort-specific neuropsychological batteries and adjudication procedures, reflecting differences in diagnostic environments and available expertise. Although diagnoses were harmonized into common analytic categories for comparative analyses, residual variability in case ascertainment and diagnostic thresholds may persist. Such heterogeneity may influence cross-cohort comparisons of cognitive impairment prevalence but is unlikely to explain the consistent patterns of CMRFs underdiagnosis observed across settings. We conducted multiple statistical tests across cohorts and outcomes. Given the exploratory and hypothesis-generating nature of these analyses, we did not apply formal correction for multiple comparisons. Accordingly, findings, particularly those near conventional significance thresholds, should be interpreted cautiously and viewed as provisional pending replication. Recruitment periods, diagnostic environments, healthcare access, and measurement protocols differed across cohorts, with enrollment spanning multiple decades. Improvements in cardiometabolic screening practices over time and variation in clinical infrastructure may have influenced observed rates of underdiagnosis. Accordingly, cross-cohort comparisons should be interpreted as context-specific and descriptive rather than strictly comparable across time and healthcare systems. Sample sizes in some of the cohorts had limited power for subgroup analyses. Nevertheless, the consistency of underdiagnosis across settings supports the robustness of the central findings. Data on antihypertensive, glucose-lowering, and psychotropic medications were collected inconsistently across cohorts and were not harmonized for pooled analyses; as a result, these variables could not be included as covariates in adjusted models, representing a potential source of residual confounding. For the hypertension underdiagnosis outcome, we used an SBP‑only operational definition (SBP ≥ 130 mmHg without self‑reported hypertension) to ensure cross‑site harmonization with single time‑point measurements. This approach may undercapture isolated diastolic hypertension and thus represents a potential source of misclassification. Because awareness was assessed by self‑report, some misclassification is possible, particularly in subgroups with limited health literacy. However, self‑report directly captures the construct of personal awareness central to our definition of underdiagnosis, whereas medical records index documentation or treatment exposure rather than individual awareness.

Future studies should extend these analyses longitudinally to determine whether underdiagnosed cardiometabolic risk predicts subsequent cognitive decline and incident dementia and whether improving detection alters dementia trajectories. Additionally, integration of neuroimaging, blood-based biomarkers, and digital cognitive assessments may help clarify mechanisms linking unrecognized cardiometabolic burden to neurodegeneration. Lastly, expanding cohort representation across African regions and diaspora contexts will further inform region-specific prevention strategies and embedding such work within emerging African brain health networks, including AFRICA-FINGERS^34^, FeMBER-AFRICA^35^, the African Dementia Consortium^36^, and the Global Brain Health Institute^37^, offers a pathway toward sustainable, equity-focused dementia prevention.

In conclusion, underdiagnosis of dementia-related CMRFs, specifically, hypertension and abnormal glycaemia is widespread across indigenous African and African diaspora populations and varies systematically by sociodemographic, geographic and healthcare contexts. These diagnostic gaps undermine dementia prevention strategies that depend on early identification and management of modifiable risk factors. Prioritizing the identification and management of cardiometabolic underdiagnosis through contextually tailored, gender-sensitive, and health system-oriented strategies is essential for promoting global brain health equity and mitigating the burden of dementia.

## Methods

### Study design and cohorts

This cross-sectional analysis included four cohorts of older adults (N = 7000) to examine underdiagnosis of CMRFs across Indigenous African and African diaspora populations. Participants were drawn from community-based studies conducted in Nigeria, the United States, and Kenya.

The Ibadan sample was drawn from the Indianapolis–Ibadan Dementia Project (IIDP) and comprised Yoruba-speaking older adults residing in a predominantly rural community in Ibadan, Nigeria. Initial recruitment began in 1992 and was followed by a second wave of recruitment in 2001 from the same community, including survivors from the first wave. The Indianapolis sample was also drawn from the IIDP and comprised African American older adults residing in an urban community in Indianapolis, Indiana. Recruitment and assessment procedures in Indianapolis paralleled those used in Ibadan, with cohort-specific adaptations for the U.S. setting. A total of 5336 IIDP participants across the two sites were included in the present study^38–40^.

The North Texas cohort comprised African American adults aged 50 years and older enrolled in the Health and Aging Brain Study–Health Disparities (HABS-HD), a community-based observational study of the long-term factors driving health disparities and progression from mild cognitive impairment to dementia, conducted in the Dallas-Fort Worth metropolitan area in Texas, United States. Enrollment of African American participants began in 2020, and a total of 1213 participants were included in the present study^41^.

The Kenyan sample was drawn from two community-based studies: Brain Resilience Kenya (BRK) and AD-DETECT Kenya, which recruited participants residing in Nairobi and surrounding urban areas. BRK enrolled adults aged 35 years and older and focuses on the physical, social, and psychological determinants of resilient aging^42,43^. AD-DETECT recruited older adults across the cognitive spectrum and focuses on developing and validating diagnostic biomarkers for dementia research in Kenya^44^. Both studies are conducted by the same investigators, and share common assessment and data collection protocols. A total of 443 participants were included from the two Kenyan studies.

#### Ethics approval, consent and data use

All studies obtained informed consent from participants and adhered to local ethical guidelines. Ethical approval for the AD-DETECT and BRK studies were obtained from the Aga Khan University Institutional Scientific and Ethics Review Committee (AD-DETECT: 2023/ISERC-135; BRK: 2023/ISERC-125) and the National Commission for Science, Technology, and Innovation (AD-DETECT: NACOSTI/P/24/33491; BRK License #: NACOSTI/P/25/418163). The HABS-HD study is conducted at The University of North Texas Health Science Center, Fort Worth, USA, under the approval of the North Texas Regional Institutional Review Board (NTR-IRB) approved protocol #2012-083. The Ibadan/Indianapolis Dementia Project was approved by the Ethics Committee of the University of Ibadan/University College Hospital, Ibadan, Nigeria as well as by the Indiana University-Purdue University of Indianapolis Institutional Review Board.

A data use agreement was established between each study host institution and the Wake Forest University Department of Epidemiology to permit access to de-identified and anonymised data analyzed in this study. Detailed descriptions of study design, clinical assessments, biomarker collection, and cardiometabolic measurements for each cohort have been published previously and are cited here for completeness^38–44^. Brief summaries of the measures relevant to the present analyses are provided below.

### Objective CMRFs measurements

Objective blood pressure measurements were obtained using appropriately sized cuffs in accordance with each study’s standardized protocol, with participants seated or supine as specified by the cohort. Systolic (SBP) and diastolic blood pressure (DBP) were recorded and averaged when multiple readings were available. For reference, blood pressure categories followed the American Heart Association guideline definitions: Stage 1 hypertension corresponds to SBP 130–139 mmHg or DBP 80–89 mmHg, and Stage 2 to SBP ≥ 140 mmHg or DBP ≥ 90 mmHg^45^. For primary analyses, we used an SBP-only definition (SBP ≥ 130 mmHg without self-reported hypertension) to ensure cross-site harmonization and consistency with single time-point measurements. For severe elevation, we used SBP ≥ 180 mmHg as an SBP‑only definition of marked systolic elevation, noting that clinical crisis classifications typically use SBP ≥ 180 mmHg or DBP ≥ 120 mmHg and require assessment of target organ damage, which was not collected^45^. This SBP-only severe threshold is presented as an epidemiologic marker rather than a clinical crisis category. Similarly, blood glucose measurements across individual study cohorts were obtained following an overnight fast and have been described in each study’s protocol. For our primary analyses, abnormal glycaemia was defined as fasting blood glucose (FBG) ≥ 100 mg/dL, which includes prediabetes (100–125 mg/dL) and diabetes ( ≥ 126 mg/dL)^46^ .

### Self-reported CMRFs diagnosis

In each cohort, participants were asked whether a doctor or health professional had ever told them they had hypertension or diabetes. Responses were recorded as binary indicators (yes or no). Participants reporting a prior diagnosis or current use of condition‑specific medications were classified as diagnosed/aware. Those reporting no diagnosis and no medication use were classified as not aware of the condition.

### Operationalization of CMRFs underdiagnosis

Underdiagnosis was defined as objectively elevated cardiometabolic risk in participants who lacked awareness of the corresponding condition (as defined above). Underdiagnosed hypertension was defined as SBP ≥ 130 mmHg without self‑reported hypertension. Underdiagnosed abnormal glycaemia was defined as FBG ≥ 100 mg/dL without self‑reported prediabetes or diabetes.

Participants with objective values below these thresholds or those reporting a prior diagnosis were not considered underdiagnosed. Individuals receiving treatment for hypertension or diabetes were classified as diagnosed regardless of their current measurements, ensuring that underdiagnosis captured lack of awareness rather than medication‑related normalization of clinical values.

### Cognitive status

Cognitive diagnoses in each cohort were assigned using established diagnostic criteria and cohort-specific adjudication procedures^38–44^. In IIDP, dementia diagnoses required concordance across diagnostic frameworks, with final classification determined through clinical review by experienced clinicians familiar with the local cultural context.

In HABS-HD, cognitive diagnosis was determined through a structured algorithm followed by expert review, with cases meeting criteria for cognitive impairment or dementia and a subset of cognitively unimpaired participants undergoing detailed chart review and consensus adjudication. Diagnoses incorporated neuropsychological performance, clinical information, and functional status, and were assigned based on established criteria for dementia.

In the Kenyan studies, cognitive status was assigned using neuropsychological testing and clinical evaluation according to locally implemented protocols aligned with international diagnostic standards.

For the present analyses, cohort-specific diagnoses were first mapped into three harmonized categories: cognitively normal (N), cognitively impaired but not demented (CI), and dementia (D). To increase sample size and statistical power for selected analyses, we created a second cognitive variable by collapsing CI and D into a single category (cognitive impairment, CI) and contrasting it with no cognitive impairment (NCI).

### Plasma Alzheimer’s disease-related biomarkers

In the North Texas cohort, plasma biomarkers were assayed using single molecule array (Simoa) technology on the Quanterix HD-X platform following the study’s standard laboratory procedures. Analytes included plasma amyloid beta (Aβ) 40 and 42, the Aβ42/Aβ40 ratio, glial fibrillary acidic protein (GFAP), neurofilament light chain (NfL), and phosphorylated tau (pTau181 and pTau217). All biomarker values were log_10-_transformed and then standardized to reduce skewness and place analytes on a common scale prior to downstream analyses.

### Covariates

Demographic covariates were harmonized across cohorts. Age was treated as a continuous variable in years. Sex was coded as female or male. Education was dichotomized as below secondary school education (i.e., <12 years of schooling) versus secondary school or higher ( ≥ 13 years of schooling) to account for differences in educational systems and distributions across sites. Marital status was categorized as married versus not married.

APOE ε4 status was included as a genetic covariate and categorized as carrier (one or more ε4 alleles) versus non-carrier. APOE genotyping was conducted using cohort-specific protocols, and ε4 status was harmonized across studies for pooled analyses.

Socioeconomic position was operationalized using cohort-specific indicators. In the North Texas cohort, socioeconomic disadvantage was captured using a Socioeconomic Disadvantage Index (SDI) derived from five indicators: low income (<$49,999), insurance vulnerability (Medicaid or uninsured), recent medical hardship (inability to afford or access healthcare in the past 12 months), housing vulnerability (renting or housing other than home ownership), and educational vulnerability (below high school education). Each indicator contributed one point, with SDI scores ranging from 0 to 5 and higher scores indicating greater disadvantage.

In the Kenya cohort, socioeconomic deprivation was characterized using a Multidimensional Poverty Index (MPI)–based measure derived from household and individual deprivation indicators, including lack of formal education, low income, and limited access to basic utilities or sanitation^47^. The MPI was treated as a continuous variable, with higher values indicating greater deprivation. In the Ibadan and Indianapolis cohorts, sufficient information to derive composite socioeconomic measures was not available, and education was therefore used as a proxy for socioeconomic position.

Data from all cohorts were cleaned and harmonized prior to analysis. Variables were recoded to ensure comparability across studies, and cohort-specific cognitive diagnoses were mapped into shared analytic categories. Variables unavailable in specific cohorts (SDI, MPI, or APOE ε4 status) were excluded from those cohort-specific analyses.

Participants missing key measures were excluded from condition-specific analyses. No statistical imputation was performed. Blood pressure and self-reported diagnosis data were available for all included participants, while fasting glucose in the Ibadan cohort was collected in a subset and analyses of glycaemia were restricted to that subset.

### Statistical analysis

All analyses were conducted using R (version 4.0 or later). Descriptive statistics were generated separately for each cohort, with continuous variables summarized using means and standard deviations and categorical variables using proportions. Cross‑cohort comparisons used one‑way ANOVA for continuous variables and Pearson’s χ² tests for categorical variables.

Cross-sectional associations between cardiometabolic risk factors (hypertension and abnormal glycaemia) and cognitive status were examined using cohort-stratified logistic regression models. The primary cognitive outcome was any cognitive impairment, defined by collapsing cognitively impaired but not demented (CI) and dementia (D) into a single category and contrasting it with no cognitive impairment (NCI). Odds ratios (ORs) with 95% confidence intervals (CIs) were reported. Supplementary analyses modelled dementia as dementia (D) versus cognitively normal (N), excluding CI.

In the North Texas cohort, we additionally examined associations between cardiometabolic disease severity and plasma AD-related biomarkers. Separate models were fitted for early-stage disease (SBP ≥ 130 mmHg; FBG ≥ 100 mg/dL) and clinically overt disease (SBP ≥ 180 mmHg; FBG ≥ 126 mg/dL). Each biomarker was modelled as a continuous outcome with adjustment for age, sex, APOE ε4 status, and cognitive status (CI vs NCI), to assess whether associations with cardiometabolic disease were present independent of clinical cognitive status. Analyses were restricted to participants with available biomarker and cardiometabolic data.

Primary analyses examined the underdiagnosis of hypertension and abnormal glycaemia. Underdiagnosis prevalence was calculated within each cohort by comparing objectively measured cardiometabolic risk status with self‑reported diagnosis. Differences in underdiagnosis rates across cohorts were evaluated using chi‑squared tests.

To identify predictors of underdiagnosis, we fit cohort‑specific logistic regression models. For each cohort, predictors were evaluated in separate (univariate) models that were additionally adjusted for APOE ε4 status, except when APOE ε4 was the predictor of interest. This approach allowed associations to vary across social and healthcare contexts while preserving sample size in the presence of variable‑specific missingness across cohorts. Covariates (when examined as predictors in their own models) comprised age, sex, education, marital status, cognitive status, and APOE ε4 status; in the North Texas and Kenya cohorts, SDI and MPI were additionally examined to reflect context‑specific socioeconomic and material disadvantage, respectively. Where applicable, Firth’s penalized likelihood logistic regression was used to mitigate small-sample bias and potential separation. Because models were estimated separately for each predictor, analytic sample size (N) varies by predictor and reflects available‑case analysis for that predictor and APOE ε4 (when applicable).

All statistical tests were two‑sided with a significance threshold of α = 0.05. Associations with p‑values between 0.05 and 0.10 were considered exploratory trends, with interpretation emphasizing consistency and substantive relevance rather than marginal significance. Because multiple tests were conducted across cohorts and outcomes and analyses were hypothesis‑generating, no formal correction for multiple comparisons was applied; borderline findings were interpreted cautiously.

## Supplementary information

Supplementary Material_5_1_26

## Data Availability

The data supporting the findings of this study are available from contributing studies including the Indianapolis-Ibadan Dementia project (IIDP), Brain Resilience Kenya (BRK), AD-Detect Kenya, and the Health and Aging Brain Study–Health Disparities (HABS-HD). Access to IIDP, BRK and AD-Detect is subject to approval by the respective study Principal investigators and ethics committees. HABS-HD data are available through the Laboratory of Neuro Imaging (LONI) Image and Data Archive (https://ida.loni.usc.edu/login.jsp) under controlled access.

## Code availability

The codes used to generate the analyses in this study are available from the corresponding author on reasonable request.
