# Supplementary material for: Contextual variability in under-diagnosed cardiometabolic disease and cognitive vulnerability among populations at high risk for Alzheimer’s disease and related dementias
Source: NPJ Dement. 2026 Jun 19;2(1):48. doi: 10.1038/s44400-026-00099-3 (PMC13282169; doi:10.1038/s44400-026-00099-3)
Supplement: Supplementary file 1 — Supplementary Material_5_1_26 [file 44400_2026_99_MOESM1_ESM.pdf]

Supplementary Information

Table S1 | Pairwise comparisons of cohort cardiometabolic characteristics to assess between-cohort differences

Variable	Comparison	Contrast Value	P Value
Age	Indianapolis - Ibadan	1.31	<0.001
Age	Kenya - Ibadan	-20.30	<0.001
Age	Kenya - Indianapolis	-21.61	<0.001
Age	Kenya - North Texas	-6.61	<0.001
Age	North Texas - Ibadan	-13.69	<0.001
Age	North Texas - Indianapolis	-15.00	<0.001
Systolic Blood Pressure	Indianapolis - Ibadan	-5.96	<0.001
Systolic Blood Pressure	Kenya - Ibadan	-17.21	<0.001
Systolic Blood Pressure	Kenya - Indianapolis	-11.25	<0.001
Systolic Blood Pressure	Kenya - North Texas	-2.24	0.115
Systolic Blood Pressure	North Texas - Ibadan	-14.97	<0.001
Systolic Blood Pressure	North Texas - Indianapolis	-9.01	<0.001
Fasting Blood Glucose	Indianapolis - Ibadan	32.21	<0.001
Fasting Blood Glucose	Kenya - Ibadan	11.45	<0.001
Fasting Blood Glucose	Kenya - Indianapolis	-20.75	<0.001
Fasting Blood Glucose	Kenya - North Texas	-9.38	<0.001
Fasting Blood Glucose	North Texas - Ibadan	20.83	<0.001

Fasting Blood Glucose	North Texas - Indianapolis	-11.38	<0.001

p-values are from pairwise comparisons between cohorts using t-tests or χ^2 tests, as appropriate.

Table S2 | Associations between hypertension and dementia diagnosis by cohort

Location	Outcome Contrast	Predictor	Sample Size (N)	Odds Ratio	95% CI Lower	95% CI Upper	P Value
Ibadan	D vs N	Hypertension (SBP ≥ 130)	327	1.24	0.55	2.81	0.601
Indianapolis	D vs N	Hypertension (SBP ≥ 130)	226	0.83	0.38	1.81	0.635
North Texas	D vs N	Hypertension (SBP ≥ 130)	860	1.07	0.62	1.83	0.814
Kenya	D vs N	Hypertension (SBP ≥ 130)	410	0.92	0.36	2.30	0.851

Models are cohort-stratified logistic regression models adjusted for age and sex. Odds ratios (ORs) and 95% confidence intervals are shown. *N* normal cognition, *D* dementia.

Table S3 | Associations between abnormal glycemia and dementia diagnosis by cohort

Location	Outcome Contrast	Predictor	Sample Size (N)	Odds Ratio	95% CI Lower	95% CI Upper	P Value
Ibadan	D vs N	Prediabetic/Diabetic (FBG ≥ 100)	149	0.00	0.00	*Inf	0.993
Indianapolis	D vs N	Prediabetic/Diabetic (FBG ≥ 100)	170	1.25	0.52	2.98	0.621
North Texas	D vs N	Prediabetic/Diabetic (FBG ≥ 100)	798	1.63	0.95	2.81	0.077
Kenya	D vs N	Prediabetic/Diabetic (FBG ≥ 100)	397	2.85	1.05	7.79	**0.041**

Models are cohort-stratified logistic regression models adjusted for age and sex.
Abnormal glycemia is defined as fasting blood glucose (FBG) ≥ 100 mg/dL. Odds ratios (ORs) and 95% confidence intervals (CI) are shown. *N* normal cognition, *D* dementia.
Bolded *p* values are statistically significant at $p < 0.05$.
*In Ibadan, sparse data resulted in quasi-complete separation and an infinite upper CI in a firth penalized regression model.

Table S4 | Distribution of Alzheimer's disease-related biomarkers in North Texas cohort

Variable	N	Mean (SD)
Aβ42	717	8.18 (2.9)
Aβ40	703	198.47 (115.59)
Aβ42/Aβ40 ratio	702	0.04 (0.01)
GFAP	656	186.15 (173.07)
NfL	718	13.55 (11.89)
pTau181	727	16.52 (32.02)
pTau217	593	0.39 (0.32)

Values are shown as mean (SD); N indicates available observations. Aβ42/Aβ40 is the amyloid-β 42/40 ratio; GFAP, glial fibrillary acidic protein; NfL, neurofilament light; pTau181 and pTau217, phosphorylated tau at threonine 181 and 217.

Table S5 | Associations of cardiometabolic conditions with plasma Alzheimer's disease-related biomarkers

Cardiometabolic condition	Biomarker	β (SE)	p value
Diabetes (FBG ≥ 126 mg/dL)	Aβ42/Aβ40 ratio	−0.266 (0.142)	0.0612
	pTau217	0.00009 (0.105)	0.9993
	pTau181	−0.037 (0.107)	0.7268
	NfL	0.369 (0.100)	0.0002
	GFAP	−0.173 (0.183)	0.3443
Severe hypertension (SBP ≥ 180 mmHg)	Aβ42/Aβ40 ratio	0.060 (0.332)	0.8576
	pTau217	0.627 (0.268)	0.0199
	pTau181	0.600 (0.297)	0.0435
	NfL	0.618 (0.275)	0.0247
	GFAP	−0.113 (0.425)	0.7904

Biomarkers were log10-transformed and standardized prior to analysis. Each biomarker was analyzed in a separate model adjusted for age, sex, APOE ε4 carrier status, and cognitive status. Bolded *p* values are statistically significant at $p < 0.05$. Abbreviations Aβ4 = amyloid beta; NfL = neurofilament light; GFAP = glial fibrillary acidic protein; FBG = fasting blood glucose; SBP = systolic blood pressure.

Table S6 | Predictors of hypertension underdiagnosis in Ibadan cohort

Predictor	Sample Size (N)	Odds Ratio	CI Lower	CI Upper	P Value
Age	1307	1.00	0.98	1.02	0.752
Sex	1307	1.14	0.90	1.44	0.289
Education	1307	2.57	0.95	7.41	0.062
Marital status	1307	1.32	1.05	1.65	**0.017**
Cognitive status	323	1.50	0.97	2.34	0.071
APOE4 status	1307	0.79	0.63	0.99	**0.043**

Odds ratios (ORs) and 95% confidence intervals (CIs) are estimated from cohort-specific logistic regression models in which each predictor was evaluated in a separate model. All models are adjusted for APOE ε4 status except when APOE ε4 is the predictor. Sample size (N) reflects complete data for the outcome, the predictor, and APOE ε4 (when applicable), and therefore varies across predictors. Hypertension was defined as SBP ≥130 mmHg; underdiagnosis was defined as SBP ≥130 mmHg without a self-reported hypertension diagnosis. Reference groups: Sex = Male; Education = Secondary school or above; Marital status = Married; Cognitive status = No cognitive impairment; APOE ε4 = Non-carrier. $P < 0.05$ is bolded.

Table S7 | Predictors of hypertension underdiagnosis in Indianapolis cohort

Predictor	Sample Size (N)	Odds Ratio	CI Lower	CI Upper	P Value
Age	1180	0.97	0.94	1.00	**0.025**
Sex	1180	0.59	0.44	0.79	**0.000**
Education	1178	0.89	0.67	1.18	0.425
Marital status	1179	0.79	0.59	1.05	0.101
Cognitive status	200	0.64	0.30	1.35	0.241
APOE4 status	1180	1.29	0.97	1.73	0.081

Odds ratios (ORs) and 95% confidence intervals (CIs) are estimated from cohort-specific logistic regression models in which each predictor was evaluated in a separate model. All models are adjusted for APOE ε4 status except when APOE ε4 is the predictor. Sample size (N) reflects complete data for the outcome, the predictor, and APOE ε4 (when applicable), and therefore varies across predictors. Hypertension was defined as SBP ≥130 mmHg; underdiagnosis was defined as SBP ≥130 mmHg without a self-reported hypertension diagnosis. Reference groups: Sex = Male; Education = Secondary school or above; Marital status = Married; Cognitive status = No cognitive impairment; APOE ε4 = Non-carrier. $P < 0.05$ is bolded.

Table S8 | Predictors of hypertension underdiagnosis in North Texas cohort

Predictor	Sample Size (N)	Odds Ratio	CI Lower	CI Upper	P Value
Age	626	0.97	0.94	0.99	**0.004**
Sex	626	0.80	0.56	1.14	0.216
Education	626	0.84	0.43	1.65	0.613
Marital status	624	1.12	0.79	1.60	0.520
Cognitive status	625	0.94	0.65	1.35	0.721
APOE4 Status	626	0.86	0.60	1.23	0.402
SDI	579	1.00	0.83	1.21	0.992

Odds ratios (ORs) and 95% confidence intervals (CIs) are estimated from cohort-specific logistic regression models in which each predictor was evaluated in a separate model. All models are adjusted for APOE ε4 status except when APOE ε4 is the predictor. Sample size (N) reflects complete data for the outcome, the predictor, and APOE ε4 (when applicable), and therefore varies across predictors. Hypertension was defined as SBP ≥130 mmHg; underdiagnosis was defined as SBP ≥130 mmHg without a self-reported hypertension diagnosis. Reference groups: Sex = Male; Education = Secondary school or above; Marital status = Married; Cognitive status = No cognitive impairment; APOE ε4 = Non-carrier. P < 0.05 is bolded.

Table S9 | Predictors of hypertension underdiagnosis in Kenya cohort

Predictor	Sample Size (N)	Odds Ratio	CI Lower	CI Upper	P Value
Age	93	0.98	0.95	1.02	0.357
Sex	93	0.60	0.26	1.37	0.221
Education	93	1.62	0.60	4.39	0.339
Marital status	93	0.58	0.24	1.37	0.213
Cognitive status	89	0.95	0.38	2.34	0.905
Multidimensional Poverty Index	93	0.79	0.53	1.18	0.257

Odds ratios (ORs) and 95% confidence intervals (CIs) are estimated from cohort-specific logistic regression models in which each predictor was evaluated in a separate model. All models are adjusted for APOE ε4 status except when APOE ε4 is the predictor. Sample size (N) reflects complete data for the outcome, the predictor, and APOE ε4 (when applicable), and therefore varies across predictors. Hypertension was defined as SBP ≥130 mmHg; underdiagnosis was defined as SBP ≥130 mmHg without a self-reported hypertension diagnosis. Reference groups: Sex = Male; Education = Secondary school or above; Marital status = Married; Cognitive status = No cognitive impairment; APOE ε4 = Non-carrier.

Table S10 | Predictors of abnormal glycemia underdiagnosis in Ibadan cohort

Predictor	Sample Size (N)	Odds Ratio	CI Lower	CI Upper	P Value
Age	83	0.89	0.79	1.01	0.073
Sex	83	0.14	0.00	1.23	0.083
Marital status	83	0.59	0.10	2.86	0.515
Cognitive status	19	11.66	0.37	2,204.75	0.168
APOE4 status	83	0.64	0.13	3.21	0.576

Odds ratios (ORs) and 95% confidence intervals (CIs) are estimated from predictor-specific Firth penalized logistic regression models. Each predictor was modeled separately and adjusted for APOE ε4 status, except when APOE ε4 was the predictor. Sample size (N) reflects complete data for the outcome, the predictor shown, and APOE ε4 (when applicable), and therefore varies across predictors. Abnormal glycaemia was defined as fasting blood glucose (FBG) ≥100 mg/dL; underdiagnosis was defined as FBG ≥100 mg/dL without a self-reported diabetes diagnosis. Reference groups: Sex = Male; Marital status = Married; Cognitive status = No cognitive impairment; APOE ε4 = Non-carrier. Due to sparse data in cognitive status categories, the corresponding estimate shows a wide CI. Education was omitted due to non-estimation.

Table S11 | Predictors of abnormal glycemia underdiagnosis in the Indianapolis cohort

Predictor	Sample Size (N)	Odds Ratio	CI Lower	CI Upper	P Value
Age	682	1.02	1.00	1.05	0.098
Sex	682	1.06	0.77	1.45	0.712
Education	682	1.01	0.74	1.36	0.973
Marital status	682	1.13	0.83	1.55	0.425
Cognitive status	122	1.62	0.79	3.35	0.189
APOE4 status	682	1.06	0.78	1.45	0.696

Odds ratios (ORs) and 95% confidence intervals (CIs) are estimated from predictor-specific logistic regression models within each cohort. Each predictor was modeled separately and adjusted for APOE ε4 status, except when APOE ε4 was the predictor. Sample size (N) reflects complete data for the outcome, the predictor shown, and APOE ε4 (when applicable), and therefore varies across predictors. Abnormal glycaemia was defined as fasting blood glucose (FBG) ≥100 mg/dL; underdiagnosis was defined as FBG ≥100 mg/dL without a self-reported diabetes diagnosis. Reference groups: Sex = Male; Marital status = Married; Cognitive status = No cognitive impairment; APOE ε4 = Non-carrier.

Table S12 | Predictors of abnormal glycemia underdiagnosis in North Texas cohort

Predictor	Sample Size (N)	Odds Ratio	CI Lower	CI Upper	P Value
Age	324	0.99	0.96	1.02	0.382
Sex	324	1.01	0.65	1.58	0.964
Education	324	0.38	0.16	0.92	**0.032**
Marital status	323	1.59	1.02	2.49	**0.041**
Cognitive status	323	0.96	0.62	1.51	0.874
APOE4 status	324	1.36	0.86	2.14	0.188
SDI	307	1.04	0.82	1.30	0.756

Odds ratios (ORs) and 95% confidence intervals (CIs) are estimated from predictor-specific logistic regression models within each cohort. Each predictor was modeled separately and adjusted for APOE ε4 status, except when APOE ε4 was the predictor. Sample size (N) reflects complete data for the outcome, the predictor shown, and APOE ε4 (when applicable), and therefore varies across predictors. Abnormal glycaemia was defined as fasting blood glucose (FBG) ≥100 mg/dL; underdiagnosis was defined as FBG ≥100 mg/dL without a self-reported diabetes diagnosis. Reference groups: Sex = Male; Marital status = Married; Cognitive status = No cognitive impairment; APOE ε4 = Non-carrier. $P < 0.05$ is bolded.

Table S13 | Predictors of abnormal glycemia underdiagnosis in Kenya cohort

Predictor	Sample Size (N)	Odds Ratio	CI Lower	CI Upper	P Value
Age	36	0.97	0.91	1.04	0.361
Sex	36	0.75	0.19	2.89	0.676
Education	36	6.07	0.66	56.03	0.112
Marital status	36	0.76	0.19	3.00	0.697
Cognitive status	32	0.33	0.07	1.52	0.156
Multidimensional Poverty Index	36	3.18	1.10	9.21	**0.033**

Odds ratios (ORs) and 95% confidence intervals (CIs) are estimated from predictor-specific logistic regression models within each cohort. Each predictor was modeled separately and adjusted for APOE ε4 status, except when APOE ε4 was the predictor. Sample size (N) reflects complete data for the outcome, the predictor shown, and APOE ε4 (when applicable), and therefore varies across predictors. Abnormal glycaemia was defined as fasting blood glucose (FBG) ≥100 mg/dL; underdiagnosis was defined as FBG ≥100 mg/dL without a self-reported diabetes diagnosis. Reference groups: Sex = Male; Marital status = Married; Cognitive status = No cognitive impairment; APOE ε4 = Non-carrier. $P < 0.05$ is bolded.
